# Oncometabolic mutation IDH1 R132H confers a metformin-hypersensitive phenotype

**DOI:** 10.18632/oncotarget.3733

**Published:** 2015-03-30

**Authors:** Elisabet Cuyàs, Salvador Fernández-Arroyo, Bruna Corominas-Faja, Esther Rodríguez-Gallego, Joaquim Bosch-Barrera, Begoña Martin-Castillo, Rafael De Llorens, Jorge Joven, Javier A. Menendez

**Affiliations:** ^1^ Metabolism and Cancer Group, Translational Research Laboratory, Catalan Institute of Oncology (ICO), Girona, Catalonia, Spain; ^2^ Molecular Oncology Group, Girona Biomedical Research Institute (IDIBGI), Girona, Catalonia, Spain; ^3^ Unitat de Recerca Biomèdica (URB-CRB), Institut d’Investigació Sanitaria Pere i Virgili (IISPV), Universitat Rovira i Virgili, Reus, Catalonia. Spain; ^4^ Medical Oncology, Catalan Institute of Oncology (ICO), Girona, Catalonia, Spain; ^5^ Clinical Research Unit, Catalan Institute of Oncology (ICO), Girona, Catalonia, Spain; ^6^ Biochemistry and Molecular Biology Unit, Department of Biology, University of Girona, Girona, Catalonia, Spain

**Keywords:** IDH1, oncometabolites, metformin, anaplerosis, cancer

## Abstract

Metabolic flexibility might be particularly constrained in tumors bearing mutations in isocitrate dehydrogenase 1 (IDH1) leading to the production of the oncometabolite 2-hydroxygluratate (2HG). To test the hypothesis that IDH1 mutations could generate metabolic vulnerabilities for therapeutic intervention, we utilized an MCF10A cell line engineered with an arginine-to-histidine conversion at position 132 (R132H) in the catalytic site of IDH1, which equips the enzyme with a neomorphic α-ketoglutarate to 2HG reducing activity in an otherwise isogenic background. IDH1 R132H/+ and isogenic IDH1 +/+ parental cells were screened for their ability to generate energy-rich NADH when cultured in a standardized high-throughput Phenotype MicroArrayplatform comprising >300 nutrients. A radical remodeling of the metabotype occurred in cells carrying the R132H mutation since they presented a markedly altered ability to utilize numerous carbon catabolic fuels. A mitochondria toxicity-screening modality confirmed a severe inability of IDH1-mutated cells to use various carbon substrates that are fed into the electron transport chain at different points. The mitochondrial biguanide poisons, metformin and phenformin, further impaired the intrinsic weakness of IDH1-mutant cells to use certain carbon-energy sources. Additionally, metabolic reprogramming of IDH1-mutant cells increased their sensitivity to metformin in assays of cell proliferation, clonogenic potential, and mammosphere formation. Targeted metabolomics studies revealed that the ability of metformin to interfere with the anaplerotic entry of glutamine into the tricarboxylic acid cycle could explain the hypersensitivity of IDH1-mutant cells to biguanides. Moreover, synergistic interactions occurred when metformin treatment was combined with the selective R132H-IDH1 inhibitor AGI-5198. Together, these results suggest that therapy involving the simultaneous targeting of metabolic vulnerabilities with metformin, and 2HG overproduction with mutant-selective inhibitors (AGI-5198-related AG-120 [Agios]), might represent a worthwhile avenue of exploration in the treatment of IDH1-mutated tumors.

## INTRODUCTION

The discovery of cancer-specific mutations in metabolic enzymes has led to a renewed interest in cancer metabolism as a potential source of new targets for preventing and treating cancer [1-14]. Loss-of-function germline mutations in tumor suppressor genes encoding the Krebs cycle enzymes fumarate hydratase (FH) and succinate dehydrogenase (SDH) have been identified in some forms of human renal cell cancer, paraganglioma, and pheochromocytoma. FH- and SDH-deficient cells and tumors exhibit activation of hypoxia-inducible factor-alpha (HIFα) under normoxia, termed pseudohypoxia, which can be attributed to the allosteric inhibition of HIFα-prolyl hydroxylases by the accumulation of fumarate and succinate, respectively [15-22]. The term oncometabolite has been coined to define a small molecule component (or enantiomer) of normal metabolism whose abnormal accumulation establishes a milieu that initiates carcinogenesis. Another example of genetic alterations in metabolic enzymes leading to accumulation of oncometabolites is the occurrence of activating oncogenic mutations in isocitrate dehydrogenase 1 and 2 (IDH1 and IDH2) producing the oncometabolite (D)-2-hydroxyglutarate (2HG), a phenomenon that has been identified in a variety of tumor types including acute myeloid leukemia (AML), glioma, chondrosarcoma, and intrahepatic cholangiocarcinoma, a deadly liver cancer [23-27].

IDH1 and IDH2 mutant enzymes lose the ability to catalyze the reversible NADP^+^-dependent conversion of isocitrate to α-ketoglutarate (αKG) in the cytoplasm or mitochondria, respectively. Additionally, mutations in IDH result in a gain-of-function activity that involves the reductive NADPH-dependent conversion of αKG to 2HG. Importantly, the 2HG-producing neomorphic activity of mutated IDH is strongly consistent with the genetic observation that mutant IDH alleles are almost always found in the heterozygous context, *i.e.,* the wild-type and mutant enzymes should work in concert to produce αKG that can be channeled to 2HG [28-34]. Indeed, metabolic profiling studies have demonstrated that cancer cells expressing IDH1 R132H, the most frequently-found mutation in IDH1 converting arginine residue 132 to histidine, accumulate extraordinarily high concentrations of 2HG (>10 mmol/L), which is in sharp contrast with the normal cellular concentration of αKG (~0.4 mmol/L). Intriguingly, although the initial connection between cancer and 2HG appeared to exclusively involve the pathological overproduction of 2HG by mutant IDHs, recent studies have demonstrated elevated levels of 2HG in biologically aggressive breast cancer tumors without IDH mutation [35]. 2HG overproduction significantly associates with c-Myc activation and poor prognosis in breast carcinomas bearing a stem cell-like transcriptional signature and overexpressing glutaminase, which suggests a functional relationship between glutamine and 2HG metabolism in breast cancer [36]. Moreover, elevated levels of 2HG in breast cancer cells without IDH mutation can be also driven by overexpression of the serine biosynthetic pathway enzyme phosphoglycerate dehydrogenase (PHGDH), which can catalyze the NADH-dependent reduction of αKG to 2HG [37].

Although 2HG has been shown to inhibit the activity of multiple αKG-dependent dioxygenases and initiates multiple alterations in cell differentiation, survival, and extracellular matrix maturation, the exact molecular pathways through which IDH1 mutations and overproduction of 2HG lead to tumor formation remain unclear. Furthermore, IDH1 mutations and 2HG exert their tumorigenic effects through mechanisms that are quite distinct from the classic oncogene addiction model exploited by tyrosine kinase inhibitors. Because 2HG overproduction appears to drive tumorigenesis and promotes transformation through a metabolic block that epigenetically impairs cellular differentiation, pharmacological reduction of 2HG levels could provide therapeutic benefit in patients with malignancies harboring gain-of-function IDH mutations. Accordingly, treatment with small-molecule inhibitors specifically targeting the R132H mutation has revealed that many of the effects of mutant IDH1, including histone hypermethylation, colony formation, and differentiation blockade, are indeed reversible [38-44]. Conversely, other studies have shown that the DNA hypermethylator phenotype associated with IDH mutations is not entirely reverted by a mutant IDH1 inhibitor, strongly suggesting that inhibitors solely targeting 2HG production might reverse some, but not all, mutant IDH1-dependent phenotypes. In this scenario, it is reasonable to propose that specific metabolic alterations such as IDH1 mutations, which result in metabolites or pathways becoming essential or limiting in cancer cells, may produce metabolic vulnerabilities for therapeutic interventions that do not necessarily require changes in 2HG levels [45-50].

To test the hypothesis that metabolic flexibility might be particularly constrained in tumor subtypes bearing IDH1 mutations and overproducing 2HG, we took advantage of an MCF10A cell line with an endogenous heterozygous knock-in of the clinically relevant R132H mutation generated *via* recombinant adeno-associated virus technology [51]. Using MCF10A IDH1 R132H/+ mutated cells and isogenic MCF10A IDH1 wild-type (WT) controls, we assessed whether IDH1-mutated cells have unique metabolic properties that distinguish them from WT counterparts. In addition, we evaluated the occurrence of “metabolic synthetic lethality” in response to a clinically-relevant inhibitor that perturbs mitochondrial metabolism, specifically the biguanide metformin.

## RESULTS

We hypothesized that exploring the clinically relevant R132H mutation in an otherwise isogenic background would be an idoneous means to determine potential metabolic “bystander signaling” imposed by gain-of-function mutations. To do this, we employed MCF10A cells, a non-transformed, near diploid, spontaneously immortalized human mammary epithelial cell line, with an endogenous heterozygous knock-in of IDH1 R132H generated *via* recombinant adeno-associated virus technology [51]. We first confirmed that intracellular levels of the oncometabolite 2HG were significantly increased in IDH1 R132H mutant-expressing MCF10A cells (hereafter termed R132H/+). Metabolomic analysis revealed a dramatic ~28-fold increase in 2HG levels in R132H/+ cells over parental isogenic WT control cells (see below), which was consistent with previous results. Therefore, heterozygous insertion of IDH1 R132H successfully generates heterozygously-expressed active mutant IDH1 capable of producing enormous amounts of 2HG.

### Metabolic fingerprinting reveals a decreased ability of R132H/+ cells to generate energy from numerous catabolic fuels

Metabolic fingerprinting with Phenotype Microarrays (PM) allows the large-scale analysis of nutrient utilization, and has been employed successfully to elucidate the phenotypic effects of single genetic alterations in microbial and mammalian cells [52-56]. We carried out global bioenergetic profiling of R132H/+ cells and isogenic WT controls by culturing them in four PM-Ms microplates in which the bottoms of the wells had been coated with carbon-energy substrates to create 367 unique culture conditions, including carbohydrates/starches, alcohols, fatty acids, ketones, carboxylic acids, amino acids, and bi-amino acids. PM-M1 contained primarily carbohydrate and carboxylate substrates, whereas PM-M2, M3, and M4 contained individual L-amino acids and most dipeptide combinations. We conducted the PM assay over a 2-day incubation period. R132H/+ and WT cells were incubated in 5% serum-containing Biolog IF-M1 medium (RPMI 1640 without glucose/glutamine) to provide all nutritional ingredients at sufficient levels other than carbon-sources, which were omitted. R132H/+ and WT cells were scanned for phenotypes by seeding cells in each well followed by the addition of Biolog's proprietary redox dye. Whereas widely-employed redox dyes, such as MTT, MTS and XTT, measure cellular reductase activities, the redox chemistry employed in the PM technology gives very little non-specific dye reduction and is strictly dependent on the presence of usable carbon-energy sources in the medium. Consequently, this redox dye approach accurately measures reductase activity due to energy (*i.e.,* NADH) producing catabolic pathways that use diverse biochemical substrates. In some wells, the cells were stimulated and in other wells cells were inhibited in their ability to generate energy-rich NADH. The NADH-dependent reduction of the redox dye brought about a color change that was then recorded in a standard microplate reader.

**Figure 1:**
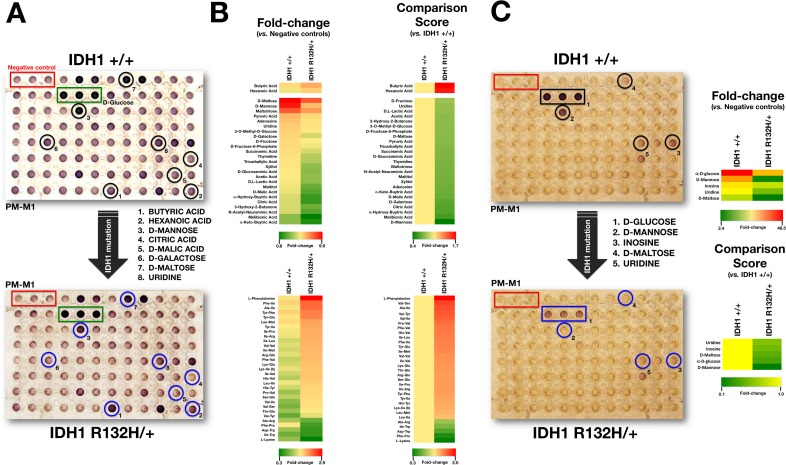
Metabolic fingerprint of the oncometabolic mutation IDH1 R132H in MCF10A breast epithelial cells **A**. Representative micrographs of non-starved IDH1 +/+ and R132H/+ cells following 48 hours culture in PM-M1 plates containing primarily carbohydrate and carboxylate substrates (see text for a detailed explanation). **B**. Phenetic maps of carbon utilization patterns of IDH1+/+ and R132H/+ cells obtained after evaluation of OD values in PM-M1, PM-M2, PM-M3, and PM-M4 plates (see text for a detailed explanation). Phenotypes that are lost are colored green and phenotypes that are gained are colored red; the exact relative values are given by a corresponding color as indicated at the color scales. **C**. Left. Representative micrographs of starved IDH1 +/+ and R132H/+ cells following 48 hours culture in PM-M1 plates containing primarily carbohydrate and carboxylate substrates (see text for a detailed explanation). Right. Phenetic maps of starved-IDH1 +/+ and R132H/+ cells were generated as shown in B.

Because the color formed from each substrate reflected the energy-producing activity of the associated catabolic pathway, as expected R132H/+ and WT cells exhibited very similar and strong reductive responses in wells containing D-glucose (Figure 1A, green boxes [positive controls]), with little or no response in wells lacking any carbon source (Figure 1A, red boxes [negative controls]). Before qualitative and quantitative analyses (see below), all wells visually resembling the negative control wells were scored as “negative”, and all wells with a noticeable purple color (greater than negative control wells) were scored as “positive”. Wells with an extremely faint color, or with small purple flecks or clumps, were scored as “borderline”. To quantitatively compare each condition rapidly and systematically, we followed the same scoring procedure as we recently employed to delineate the nutritional phenome of epithelial-to-mesenchymal (EMT)-induced cancer stem-like cellular states [57]. Briefly, we calculated the fold change in the optical density of each “positive” substrate at 590 nm that resulted from the accumulation of reduced dye over a 6-hour period after normalization of the values to those of the negative-control wells included in each of the PM-M plates (Figure 1B). To quantify these evaluations, we also calculated a comparison score from the absolute ratio between the metabolic flows of R132H/+ and WT cells occurring at the same time point (6 h) (Figure 1B).

When examining the differential ability of R132H/+ and WT cells to metabolize carbohydrate and carboxylate substrates, we observed that the R132H mutation resulted in a decreased ability to generate energy from a significant number of carbon substrates (25 out of 27, 93%). Qualitatively, it was noteworthy that the strong capability of WT cells to generate energy-rich NADH from the disaccharide D-maltose, the C-2 epimer of glucose D-mannose, and the trisaccharide maltotriose was lost in R132H/+ cells. Quantitatively, the most significant changes confirmed not only the reduced ability of R132H/+ cells to metabolize D-mannose, but also their decreased capacity to generate energy from tricarboxylic acid (TCA) cycle intermediates, including citric acid and D-malic acid, when compared with WT cells (Figure 1A, 1B, top). R132H/+ cells, however, appeared to be more competent to produce energy through oxidation of saturated short chain fatty acids, such as butyric and hexanoic (caproic) acids.

In contrast to the overall reduced capacity to catabolize carbohydrate and carboxylate substrates, R132H/+ cells exhibited notably increased catabolic responses to certain individual L-amino acids and dipeptide combinations despite a low absolute rate of dye reduction (not shown). Qualitatively, it was noteworthy that R132H/+ cells acquired an increased ability to catabolize the aromatic side chain amino acid phenylalanine and multiple dipeptides containing the branched-chain amino acid isoleucine (Figure 1B, bottom). Quantitatively, the most significant changes confirmed not only the enhanced capability of R132H/+ cells to metabolize phenylalanine, but also their increased capacity to catabolize peptides containing the branched amino acid valine (Figure 1B, bottom). Interestingly, R132H/+ cells exhibited a significantly reduced capability to catabolize the closely interconnected lysine and tryptophan amino acids relative to WT cells.

Because it could be argued that a higher background color in the negative control wells might reflect a failure of IDH1 WT cells to transition to non-glucose metabolic pathways rather than an intrinsic weakness of R132H/+ cells to employ some carbon-energy sources in the mitochondria, we decided to reevaluate the changes in nutrient utilization observed in R132H/+ cells in serum-, glucose-, and amino acid-free Biolog IF-M2 medium (RPMI 1640 without glucose/glutamine/amino acids) (Figure 1C). As the cell response to the nutrients over a 2-day incubation period mostly comprised rapid cell death, similar to the responses observed in wells with no substrates, this assay format can be considered to evaluate cell survival responses under different nutrient supply conditions. Interestingly, R132H/+ cells demonstrated a drastically reduced ability to generate NADH and survive when D-maltose, α-D-glucose, and D-mannose were the sole extracellular sources of carbon and energy (i.e., a metabolic fingerprint similar to that found in non-starved conditions) (Figure 1C). These findings, altogether, strongly support the notion that R132H/+ cells are compromised *de novo* in their ability to obtain energy from major sources of cellular carbon, a phenotype that is further exacerbated by metabolic stress.

### R132H/+ cells are drastically compromised in their ability to obtain energy from the mitochondrial electron transport chain

**Figure 2:**
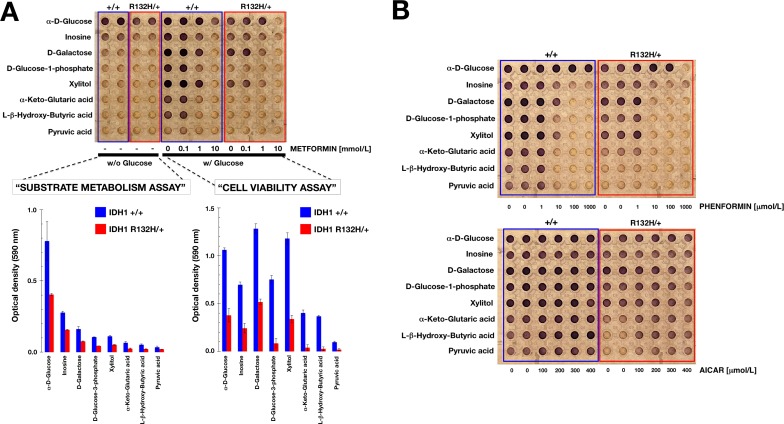
Differential mitochondrial fuel of IDH1 mutant cells **A**. Representative micrograph of the utilization patterns of 8 different mitochondrial substrates in IDH1+/+ and R132H/+ cells cultured in the absence or presence of graded concentrations of metformin for 48 h. The hexose D-galactose is metabolized to NADH *via* mitochondrial activity, whereas α-D-glucose can bypass these mitochondrial functions. Glucose-1-phosphate is metabolized differently from glucose and galactose, whereas ribose-containing inosine and xylitol are both metabolized (albeit differently) *via* the pentose phosphate pathway. αKG directly enters the TCA cycle, whereas the ketones β-hydroxybutyrate and pyruvic acid enter the TCA cycle upon metabolism and linkage to coenzyme A. **B**. Representative micrographs of the utilization patterns of 8 different mitochondrial substrates in IDH1+/+ and R132H/+ cells cultured in the absence or presence of graded concentrations of phenformin (*top*) or AICAR (*bottom*) for 48 h.

Because the above results indicated that significant remodeling of the metabotype occurs in R132H/+ cells, we employed a mitochondria toxicity-screening (PM-M TOX1) assay to evaluate the differential mitochondrial utilization of eight-carbon substrate that are fed into the ETC at different points. In the absence of exogenously-added glucose (the so-called “substrate metabolism assay”; Figure 2A), WT cells could obtain significant amounts of energy from α-D-glucose and inosine (absolute rate of dye reduction > 0.2) and very reduced amounts from substrates such as D-galactose, D-glucose-1-phosphatye and xylitol (absolute rates of dye reduction ~ 0.1). When exogenous glucose was omitted, WT cells were unable to obtain energy from α-keto-glutaric acid, DL-hydroxy-butyric acid and pyruvic acid (absolute rates of dye reduction < 0.1). In contrast, R132H/+ cells exhibited a notably reduced ability to generate energy-rich NADH from α-D-glucose and inosine and were unable to obtain significant amounts of energy from all other mitochondrial substrates (Figure 2A).

When Biolog Redox Dye MA, which contains glucose, was added to detect metabolic activity of cells that remained viable after a 48-h incubation with the 8 different carbon substrates (the so-called “cell viability assay”), WT cells were found to strongly generate energy-rich NADH following incubation with mitochondrial substrates such as inosine, D-galactose, D-glucose-1-phosphate, xylitol, α-keto-glutaric acid and L-hydroxy-butyric acid (Figure 2A). Remarkably, the ability of R132H/+ cells to generate energy-rich NADH following a long-term incubation with different mitochondrial energy sources was notably restricted to substrates D-galactose and xylitol (Figure 2A).

We further assessed the mitochondrial nature of the changes observed in the dye-reduction assay by challenging WT and R132H/+ cells cultured in the PM-M TOX1 MicroPlate with graded concentrations of the mitochondrial poison metformin. Metformin exposure blocked dye reduction of WT cells with all mitochondrial substrates in the “cell viability assay” in a dose-dependent manner (Figure 2A). Comparable results were obtained using graded concentrations of phenformin, a biguanide derivative similar to metformin (Figure 2B, top). Whereas the mitochondria-related cell viability effects of metformin and phenformin were remarkably similar, which was consistent with biguanide-related inhibition of complex I of the ETC, the selective AMPK agonist AICAR failed to recapitulate the ability of biguanides to compromise mitochondrial energy transduction and instead appeared to augment mitochondria-related cell survival in energetically stressed R132H/+ cells (Figure 2B, bottom).

### R132H mutation sensitizes breast epithelial cells to the mitochondrial poison metformin

Given that 2HG-overproducing R132H/+ cells displayed metabolic properties that distinguished them from WT isogenic cells, we wished to explore whether the increased vulnerability of IDH1 mutant cells to metformin might open a new avenue in the prevention and treatment of 2HG-overproducing IDH1-mutated tumors. To address this, we tested whether the growth properties of IDH1-mutant cells were sensitive to metformin.

**Figure 3:**
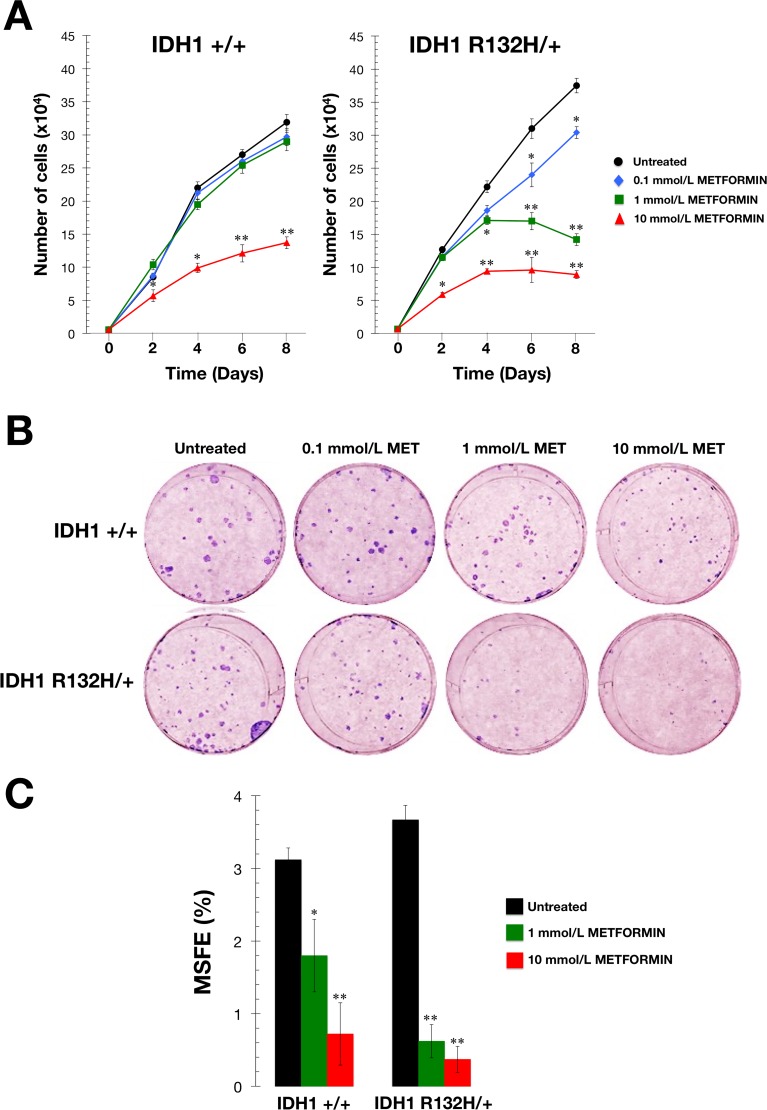
Metformin-hypersensitive phenotype of IDH1 mutant cells **A**. Cell proliferation assay. IDH1+/+ and R132H/+ cells were plated in 24-well plates at a density of 5000 cells/well and cultured in standard medium in the absence or presence of graded concentrations of metformin as specified. The data presented are the mean of cell number x 10^4^/well and SD (bars) from two independent experiments performed in triplicate and obtained after 0, 2, 4, 6, and 8 days. *P < 0.05; **P < 0.01 *versus* the corresponding controls. **B**. Clonogenic assay. Representative micrographs of crystal violet-stained colonies from IDH1+/+ and R132H/+ cells pulsed with vehicle or graded concentrations of metformin for 48 h and seeded at clonal density. **C**. Mammosphere forming assay. MSFE of IDH1+/+ and R132H/+ cells was calculated as the number of mammospheres (diameter >50 μm) formed after 7 days, divided by the original number of cells seeded and expressed as percentage mean (*columns*) ± SD (*bars*). Re-feeding of mammospheres cultures with metformin and/or sphere medium was performed on days 3 and 5. *P < 0.05; **P < 0.01 *versus* the corresponding controls.

We first measured cell proliferation in response to various concentrations of metformin over the course of 8 days. At the lowest dose of metformin tested (0.1 mmol/L), R132H/+ cells exhibited a slight decrease in growth rate compared with equivalent untreated cells, which was significant at later time periods (Figure 3A). At a dose of 1 mmol/L, metformin was ineffective against WT cells, but was sufficient to significantly perturb growth after 4 days in R132H/+ cells (Figure 3A). Additionally, 10 mmol/L metformin exerted strong cytostatic effects in R132H/+ cells, while WT cells continued to proliferate, albeit more slowly at 10 mmol/L metformin (Figure 3A).

To measure clonogenic survival, cells were treated for 48 hours with graded concentrations of metformin under adherent conditions. Following treatment, cells that remained attached were collected and seeded into 6-well plates at a density of 100 cells/well. Survival assays demonstrated a more pronounced dose-dependent inhibition of colony formation in metformin-treated R132H/+ cells when compared with metformin-treated WT cells (Figure 3B).

Finally, we used the mammosphere assay to determine whether metformin could also preferentially inhibit the self-renewal capacity of R132H/+ cells. As shown in Figure 3C, R132H/+ cells exhibited a slightly increased ability to form mammospheres in the absence of metformin compared with WT cells (3.7±0.1% versus 3.1±0.1%, respectively). Interestingly, the intrinsically enhanced spheroid formation capacity of R132H/+ cells was drastically reduced with metformin at a non-cytotoxic concentration of 1 mmol/L, which decreased mammosphere forming efficiency by >80% (Figure 3C). In contrast, an equivalent concentration of metformin reduced the mammosphere forming efficiency of WT cells by only ≈40% (Figure 3C).

### Metformin impacts glutamine metabolism via the TCA cycle in IDH1-mutant cells

**Figure 4:**
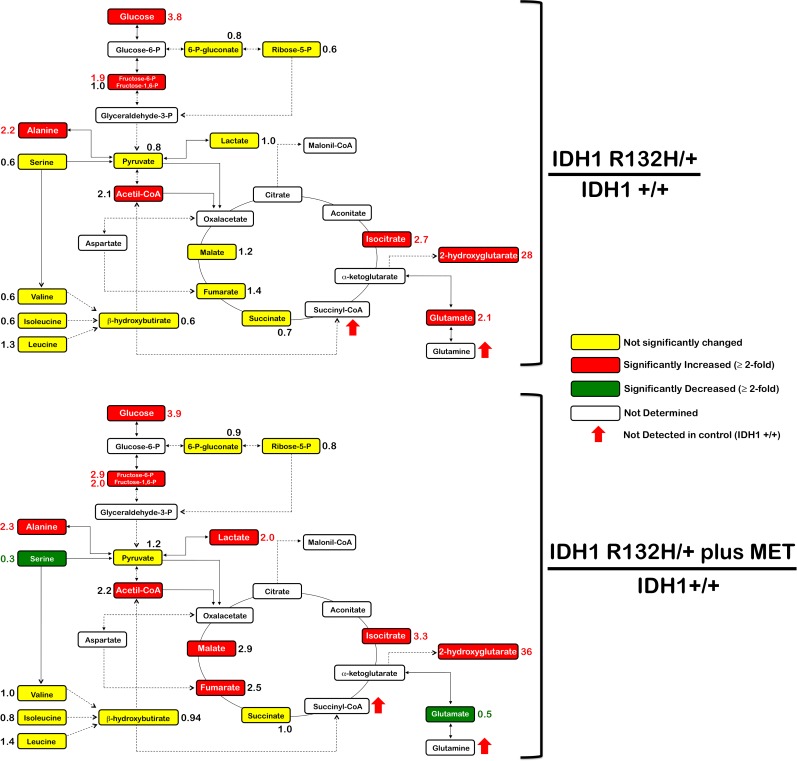
Metformin-induced metabolic changes in IDH1 mutant cells Metabolites in untreated and metformin-treated R132H/+ cells (48 h treatment, 1 mmol/L) were extracted and analyzed by GC-EI-QTOF-MS and compared with metabolites from control IDH1+/+ cells. Significantly increased and decreased metabolites are shown in red and blue colors, respectively.

To investigate whether metabolic rewiring could explain the hypersensitivity of R132H/+ cells to metformin, we used gas chromatography coupled to a quadrupole-time of flight mass spectrometer and an electron ionization interface (GC-EI-QTOF-MS) to quantitatively measure indirect markers of mitochondrial function including intermediates of glycolysis, the pentose phosphate pathway, and the TCA cycle. As expected, extremely high levels of 2HG were detected in R132H/+ cells (Figure 4). Additionally, the results of the analysis pointed to significant differences in the oxidative *versus* reductive usage of glutamine through the TCA cycle in response to metformin (Figure 4). Under baseline conditions, minimal changes were detected in the quantitative distribution of most central carbon metabolites in each cell type, with the sole exception of an activation of the glutaminolysis pathway, when considering a net accumulation of glutamate and alanine in R132H/+ cells compared with WT cells. Metformin treatment resulted in the emergence of a striking metabolic signature associated with its ability to inhibit cell growth and decrease cell survival of IDH1-mutated cells. This included substantially reduced levels of serine in R132H/+ cells. Furthermore, although metformin drastically reduced the net accumulation of cellular glutamate, indicating an inhibitory effect in the oxidative flux of glutamine, the latter half of the TCA cycle was significantly upregulated in metformin-treated R132H/+ cells as revealed by the accumulation of fumarate and malate. This finding strongly suggested that metformin treatment might decrease glucose oxidation. This effect on the oxidative metabolism of glutamine and glucose was ostensibly accompanied by a compensatory enhancement of the glycolytic flux since a significant accumulation of not only lactate, but also glycolytic intermediates such as fructose-6-phosphate and fructose-1-6-biphosphate, was evident. Importantly, metformin did not target 2HG production in R132H/+ cells since 2HG and isocitrate accumulated further relative to the baseline levels found in WT cells.

### Glutamine-related metabolic vulnerability of R132H/+ cells to metformin is independent of 2HG production

**Figure 5:**
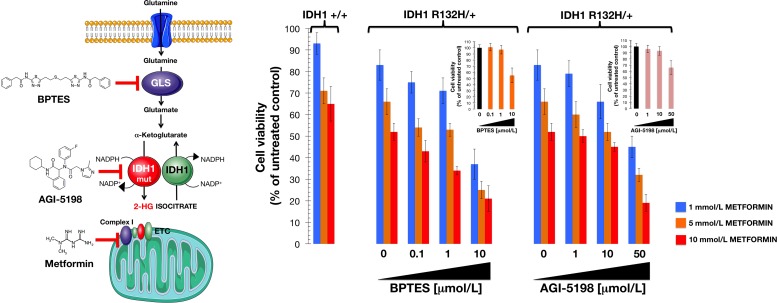
Interaction between metformin and drugs targeting the glutamine-driven production of 2HG in IDH1 mutant cells *Left.* Pathway showing production of the oncometabolite 2HG from glutamine, and inhibitor targets. GLS, glutaminase; IDH1, isocitrate dehydrogenase; IDH1 mut, mutant IDH1; ETC, electron transport chain. *Right.* Relative cell viability of cell lines treated with a combination of metformin and either the glutaminase inhibitor BPTES or the IDH1-mutant inhibitor AGI-5198. Cell viability was normalized to the condition with no metformin and no added BPTES or AGI-5198. Mean (*columns*) and SD (*bars*) were calculated from three biological replicates.

We finally questioned whether metformin's ability to obstruct glutamine metabolism could explain its apparent synthetic lethal interaction with the R132H mutation. In WT cells, inhibition of oxidative mitochondrial metabolism (e.g., by hypoxia) should limit glucose flux to mitochondria. As a response, cells will activate reductive glutamine metabolism (where glutamine is converted to acetyl-CoA via glutamate, αKG, isocitrate, and then citrate) *via* WT IDH1 to provide carbon for acetyl-CoA generation and lipid synthesis [50]. The presence of a mutant IDH1 allele will reduce glutamine metabolism. Inhibition of mitochondrial complex I by metformin has been found to decrease glucose carbon entry into the TCA cycle. This “primary effect” of metformin results in an adaptive cell response by increasing the dependency of reductive glutamine metabolism [58, 59]. To examine whether the exacerbated response of IDH1 mutant cells to metformin was related to glutamine metabolism, we treated cells with bis-2-(5-phenylacetamido-1, 2, 4-thiadiazol-2-yl) ethyl sulfide (BPTES), a selective allosteric inhibitor of glutaminase (Figure 5) [58]. Combined treatment with metformin and BPTES resulted in a larger decrease in R132H/+ cell viability than was observed when single treatments were used (Figure 5). To confirm that the metformin-hypersensitive defect of IDH1-mutated cells was a glutamine-related metabolic vulnerability that was independent of 2HG overproduction, we selectively inhibited mutant IDH1 enzyme function with AGI-5198, a cell-permeable phenyl-glycine analog that acts as an R132 somatic mutant-specific inhibitor [40]. Remarkably, co-treatment with AGI-5198 not only failed to attenuate the efficacy of metformin but instead resulted in a supra-additive (synergistic) interaction at decreasing cell viability of R132H/+ cells (Figure 5).

## DISCUSSION

We hypothesized that metabolic alterations induced by cancer-promoting IDH1 mutations might confer a distinct and therapeutically vulnerable “metabolic phenotype” that could be exploited to select drugs with the highest probability of clinical success. Because cellular context-based screens rather than the standard *in vitro* enzymatic assays are a powerful system to identify drugs that act in a particular genetic framework, we used this approach to determine metabolic differences and therapeutic potential using a “matched pair” of wild-type and isogenic IDH1-mutant MCF10A cells, a useful model to analyze the transforming effects of candidate oncogenes [65, 66]. By using a single-codon knock-in IDH1 mutation approach, we more faithfully modeled the genetic condition in human IDH1-mutated tumors compared with systems that do not have isogenic controls, or that use non-physiological mutant IDH1 expression.

Phenotype MicroArrays for Mammalian Cells (PMM) is a high-throughput technology that has been used successfully to evaluate the metabolo-phenotype of bacteria, yeast, and fungi [52-56]. We recently demonstrated the utility of this platform to characterize the nutritional phenome of EMT-driven cancer stem cell (CSC)-like phenotypes [57]. Here, we used PMM to interrogate IDH1 R132H/+ and IDH1+/+ isogenic cells using a single-assay metabolic profile of several hundred nutrient sources. PM-based global nutritional profiling revealed significant changes in energy-producing pathways upon acquisition of the clinically relevant mutation R132H in IDH1. Thus, the qualitative and quantitative differences in the usage of 367 energetic substrates provided a simple but highly informative metabolic characterization that reflected the unique bioenergetics of IDH1-mutated cells. At a global level, it was immediately evident that R132H/+ cells were much less metabolically active than WT isogenic cells in terms of the number of energy-generating carbon substrates. Thus, a single-codon mutation in IDH1 appears to cause a strong metabolic rewiring function that significantly alters entry of carbon from different nutrients for bioenergetic and proliferation-related biosynthetic processes. Moreover, these specific nutritional changes pointed to additional phenotypic alterations imposed by unique metabolic fluxes triggered by the oncometabolic mutation. In humans, maltose is cleaved by alpha-glucosidase such that two glucose molecules are available for energy production. Given that alpha-glucosidase is a resident of the endoplasmic reticulum (ER) as well as a key enzyme in the folding of nascent glycoproteins [67-69], the reduced ability of R132H/+ cells to catabolize maltose might also suggest a cell-specific alteration related to glycoprotein quality control. Additionally, R132H/+ cells exhibited a significantly decreased ability to generate energy from mannose, which is a key sugar involved in the glycolsylation and correct folding of glycoproteins. Future studies should evaluate whether IDH1 mutations can alter central carbon metabolism in a manner that ultimately impacts on the entire glycophenotype and/or induce ER stress, which may contribute to the unique pathophysiology of 2HG-overproducing IDH1-mutated tumors.

The mitochondria toxicity-screening (PM-M TOX1) method has been designed as a cell-based assay to examine the inhibitory/stimulatory effects of a particular chemical or gene mutation on energy production in a target cell line. Each of the eight PM-M TOX1 substrates (α-D-glucose, inosine, D-galactose, D-glucose-1-phosphate, xylitol, α-keto-glutaric acid, D, L-β-hydroxy-butyric acid, and pyruvic acid) is metabolized *via* one or more different pathways employing distinct cellular and mitochondrial transporters and catabolic enzymes. Because the eight PM-M TOX1 substrates utilize mitochondria to different extents and can feed into the ETC at different points, this assay provided a rapid readout of the outcomes of a single-codon knock-in IDH1 mutation with regard to the different carbon sources that can be oxidized by cell mitochondria to produce energy. The numerous bioenergetic fuel types that could not be employed by R132H/+ cells was somewhat reminiscent of the phenomenon observed in proliferating cells with dysfunctional mitochondrial oxidative phosphorylation, a metabolic feature that can be caused by 2HG itself [70-72] and also a metabolic determinant of cancer cell sensitivity to biguanides [73]. Indeed, despite the intrinsic weakness of R132H/+ cells to employ some carbon-energy sources in mitochondria, they remained sensitive to the inhibitory effects of biguanides, suggesting an inability of IDH1-mutated cells to transition their energy metabolism to the various substrates provided when challenged further with a clinically-relevant mitochondrial poison such as metformin. Moreover, the fact that R132H/+ cells acquired a generally-enhanced ability to generate NADH from numerous amino acids together with the observed effect of metformin on the levels of some intracellular metabolites, strongly supported a previously-suggested shift in the dependence of IDH1-mutated cells from glucose to glutamine [35, 36, 43, 45, 50, 63, 64] with respect to generation and usage of TCA cycle intermediates. Of note, R132H/+ cells acquired the ability to catabolize branched-chain amino acids, such as isoleucine and valine, which are known to produce an increase in glutamate by transamination with αKG. Because a remarkable depletion of the accumulated glutamate in R132H/+ cells occurred in response to cytostatic concentrations of metformin, it is reasonable to suggest that the metformin-hypersensitive phenotype of IDH1 mutated cells is related to the ability of metformin to negatively impact the enhanced engagement of IDH1-mutated cancer cells to TCA anaplerosis of glutamine.

Previous findings have shown that IDH1-mutant cells display an increased sensitivity to glutaminase inhibitors [43, 45, 63, 64]. BPTES, an allosteric inhibitor of glutaminase, was found to preferentially slow the growth of cancer cells expressing mutant IDH1 compared with those expressing WT IDH1 [45]. Similarly, we observed that the mitochondrial poison metformin preferentially reduced cell proliferation, clonogenic potential, and self-renewal capacity of mutant IDH1 cells relative to WT IDH1 cells in an isogenic context. Growth suppression by BPTES significantly lowers glutamate levels and increases glycolytic intermediates while leaving total levels of 2HG unaffected [45]. Analogous to BPTES, metformin treatment markedly depleted the intracellular pool of glutamate even though 2HG levels were not decreased but rather augmented in metformin-treated IDH1 mutant cells. Hence, the enhanced susceptibility to the growth inhibitory activity of metformin against IDH1 mutant cells was mechanistically independent of changes to 2HG production. Moreover, additive/synergistic interactions occurred when metformin was combined with either BPTES or the selective R132H-IDH1 inhibitor AGI-5198 in IDH1-mutant cells, strongly suggesting that the metformin-hypersensitive defect in IDH1-mutated cells was a glutamine-related metabolic vulnerability that was acquired independently of 2HG overproduction. A key glycolytic parameter, the lactate/pyruvate ratio, was significantly increased in metformin-treated IDH1-mutant cells but, intriguingly, metformin treatment also resulted in changes of certain TCA intermediates such as malate and fumarate. It could be argued that metformin-limited entry of pyruvate into the TCA cycle might decrease the rate at which oxaloacetate is converted to citrate and then transformed to isocitrate, which may lead to accumulation of the preceding TCA cycle intermediates malate, fumarate, and succinate. Interestingly, the levels of isocitrate were diametrically opposed in WT and IDH1-mutant cells before and after metformin treatment, with a significant accumulation of isocitrate occurring in metformin-treated IDH1-mutant cells. Moreover, levels of certain glycolytic intermediates, such as fructose-6-phosphate and fructose 1,6-biphosphate, were significantly increased with metformin treatment, indicating that glycolytic flux was altered, again partially mimicking the effects of BPTES, which preferentially impaired cell growth and induced a significant accumulation of citrate/isocitrate in IDH1-mutant cells but not in WT cells. BPTES treatment, however, decreased the TCA cycle intermediates succinate and malate [45]. Although the basis for these differences is not fully understood and the dissection of these mechanisms is beyond the scope of the current study, it is clear that metformin treatment mimics BPTES to impact the “addiction” of IDH1-mutated for glutamine anaplerosis of the TCA cycle. Unlike BTPES, however, metformin treatment further provokes changes in the coupling between glycolysis and glucose oxidation. Although our present study confirmed earlier data published by Cheong et al. [74] showing that metformin and *bona fide* AMPK agonists such as AICAR lead to markedly different biological outcomes (i.e., metformin effects might reflect sustained bioenergetics stress due to failure of mitochondrial compensation whereas AICAR could promote cancer cell survival in energetically stressed conditions), it is possible that in addition to the adaptive response in glutamine metabolism described here in response to metformin, energy homeostasis regulation by AMPK-activated 6-phosphofructo-2-kinase/fructose 2,6-bisphosphatase/PFKFB3 family of glycolytic regulators following metformin treatment might explain the increase in fructose 1,6-biphosphate [75].

**Figure 6:**
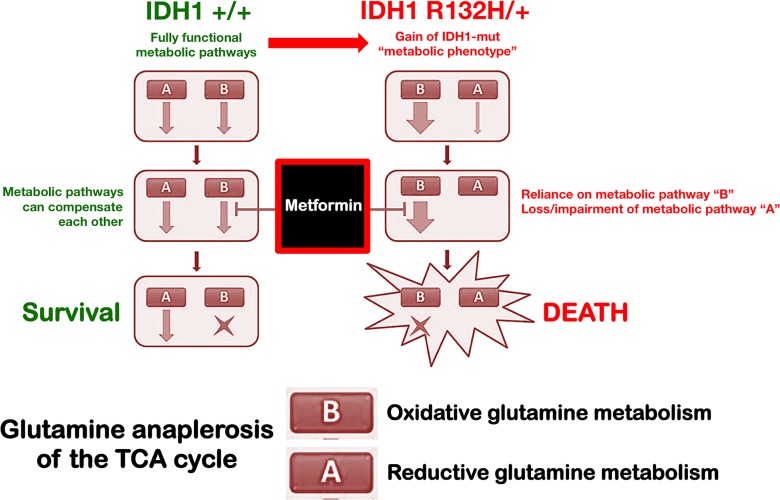
Metabolic synthetic lethality between metformin and the oncometabolic mutation IDH1 R132H IDH1 mutant cells exhibit unique metabolic properties that specifically distinguish them from the IDH1-wild type counterparts. We substantiate experimentally the fact that the cancer-driving R132H mutation drastically increases cancer cells’ vulnerability to the biguanide metformin. The ability of metformin to impact on the dependency on glutamine anaplerosis of the TCA cycle is synthetically lethal in IDH1 mutant cells, which are defective in reductive glutamine metabolism. The exacerbated metabolic vulnerability of cells bearing the 2HG-overproducing R132H mutation to clinically available biguanides may represent a new avenue in the treatment of IDH1-mutated tumors, combining clinically available metabolic drugs targeting mitochondria (e.g., metformin) and the aberrant gain-of-function of IDH1 mutant protein (e.g., AG-120).

Because glutaminase catalyzes the conversion of glutamine to glutamate, and the increased activity of this enzyme is at least partially responsible for elevated glutamine metabolism in cancer, including IDH1-mutated tumors [43, 45, 49, 60-64], the fact that BPTES-induced blockade of glutamine flux augmented the sensitivity of IDH1-mutated cells to metformin was consistent with the changes in glutamine metabolism as an adaptive response following metformin treatment. Accordingly, metformin-induced inhibition of the ETC has been reported to result in an adaptive increase in glutamine metabolism that involves a switch from oxidative to reductive pathways [58], *viz*. the conversion of glutamine to acetyl-CoA for biosynthetic processes, which ultimately leads to a dramatic decrease in ATP production by the TCA cycle. Given that mutations in IDH1 alter TCA metabolism by targeting a critical step in reductive glutamine metabolism to drastically reduce the ability of IDH1-mutant cells to fully induce this pathway, it is tempting to suggest that the ability of metformin to interfere with the anaplerotic entry of glutamine into the TCA cycle results in a synthetically lethal interaction in these cells (Figure 6). Of note, these cells are exquisitely sensitive to deficiencies in the production of glutamine-derived metabolites but are unable to mount an adaptive response to metformin because of impaired glutamine metabolism [50, 58, 59]. Moreover, an enhanced conversion of glutamine to glutamate drives the serine biosynthetic pathway (SBP), which has been shown to confer a growth advantage to tumor cells beyond providing serine and glycine for biosynthesis reactions. The SBP intersects glutaminolysis and provides not only an alternate pathway for αKG production for mitochondrial metabolism, but it also provides substrates for production of reduced glutathione (GSH), a key cellular antioxidant [76-79]. The significant and concurrent depletion of glutamate and serine provoked by metformin suggests that the metformin-induced metabolic switch might drastically and distinctively alter a response against oxidative stress. Zaprinast, a recently identified glutaminase inhibitor that is highly effective against glutamine-addicted and IDH1-mutant cells, induced significant reductions in cellular pools of glutamate, increased reactive oxygen species formation, and increased susceptibility to oxidative damage [43]. Future studies should determine whether the reported ability of metformin to inhibit glutaminase activity [80], can translate into an increased reliance on wild-type IDH1 TCA activity in IDH1-mutant cells to impair the glutamate/serine-driven maintenance of redox balance. Nonetheless, serine is also involved in folate-mediated one carbon metabolism by acting as a methyl group donor to convert tetrahydrofolate (THF) to methylene-THF, a key intermediate that fuels nucleotide metabolism and methylation reactions [81-83]. Given that metformin-impaired THF-driven one carbon metabolism [84] has been shown to strongly deplete nucleotide triphosphates and impede nucleotide synthesis in mammosphere-derived breast CSCs [85], it might be pertinent to evaluate whether metformin-induced starvation of serine underlies the strong ability of metformin to suppress the intrinsically enhanced self-renewal activity of mammosphere-initiating IDH1-mutant cells. Moreover, glutamine is a required substrate for three enzymes involved in the *de novo* synthesis of purine nucleotides and two enzymes involved in the *de novo* synthesis of pyrimidine nucleotides [86]. Therefore, glutamine-addicted IDH1-mutant cells will be expected to exhibit increased levels of nucleotides when compared to IDH1-WT cells. Preliminary experiments in our laboratory have likewise confirmed that a significant overproduction of pyrimidines takes place in R132H/+ cells, a phenomenon that was fully prevented in the presence of metformin (unpublished observations). Although we acknowledge that the current study does not address the efficacy of metformin against cancer cell models naturally bearing clinically relevant IDH1 mutations, our findings provide a direct mechanistic link to the association between metformin use and a significant reduction in incidence of intrahepatic cholangiocarcinoma (IHCC) in patients with diabetes [87]. Because IHCC is a deadly liver malignancy in which highly prevalent IDH1 mutations subvert the hepatocyte differentiation/quiescence program to create a persistent pre-neoplastic state [27], it might be tempting to suggest that chronic exposure to metformin might create a state of intolerable metabolic stress for stem-like pre-malignant cells, and thereby stopping IHCC before it starts.

In conclusion, using matched cell lines that differ only in the disease-causing gene, we here confirm that IDH1 mutant cells exhibit unique metabolic properties that distinguish them from IDH1-wild type counterparts in an isogenic context [50]. Our results confirm and expand earlier studies describing how metabolic reprogramming in mutant IDH1 cancer cells translates into changes on the cellular metabolome beyond the overproduction of the oncometabolite 2HG [88, 89]. Importantly, we demonstrate that the “metabolic phenotype” of IDH1 mutant cells results in unanticipated metabolic vulnerabilities that could be exploited for therapeutic intervention since the R132H mutation altered bioenergetic and biosynthetic demands sufficiently to strongly sensitize IDH1 mutant cells to the anti-tumoral effects of metformin. In light of these findings, it is suggested that metformin's ability to selectively reduce growth, survival, and self-renewal in cells with the oncometabolic R132H mutation may represent a new avenue in the treatment of IDH1-mutated tumors, particularly as part of a more comprehensive strategy that involves simultaneous targeting using biguanides that are already in the clinic (e.g., metformin), with mutant-selective inhibitors of IDH1 that are currently in development (e.g. AG-120 [Agios], ML309) to block production of 2HG [49]. Our current findings constitute the first direct demonstration that metabolic reprogramming driven by specific oncometabolic mutations, such as those in IDH1, can generate physiological scenarios in which the function and fate of tumor cells can be pharmacologically reshaped exclusively through metabolo-epigenetic means.

## MATERIALS and METHODS

### Drugs

Metformin (1,1-dimethylbiguanide hydrochloride) and bis-2-(5-phenylacetamido-1,2,4-thiadiazol-2-yl)ethyl sulfide (BPTES) were purchased from Sigma-Aldrich (St. Louis, MO, USA). Metformin was dissolved in sterile water to make a 1 M stock solution. AGI-5198 was purchased from Merck Millipore and dissolved in DMSO to make a 10 mmol/L stock solution.

### Cell Lines

X-MAN™ isogenic cell lines were obtained from Horizon Discovery Ltd (http://www.horizondiscovery.com). The X-MAN™ isogenic cell line MCF10A R132H/+ heterozygous knock-in of IDH1 dominant-negative R132H point mutation (HD101-013), was used in this study. The parental cell line, MCF10A IDH1 +/+ served as a control. Cells were maintained according to the supplier's recommendations (i.e., DMEM/F-12 including 2.5 mmol/L L-glutamine and 15 mmol/L HEPES, supplemented with 5% horse serum, 10 μg/mL insulin, 20 ng/mL hEGF, 0.5 μg/mL hydrocortisone, 0.1 μg/mL cholera toxin)

### Metabolic fingerprinting

MCF10A R132H/+ and MCF10A IDH1 +/+ cells (50 μL per well, 20,000 cells) were cultured in Biolog IF-M1 medium (RPMI-1640 phenol red-free medium without glucose/glutamine and containing 5% serum). This medium provided all nutritional ingredients at sufficient levels other than major C-sources, which were omitted. Cells were dispensed into Phenotype MicroArrays PM-M1, PM-M2, PM-M3, and PM-M4 (Biolog, Hayward, CA) containing 367 biochemical substrates that could potentially be metabolized and provide energy for cells. Alternatively, suspensions of MCF10A R132H/+ and MCF10A IDH1 +/+ cells in RPMI-1640 that lacked phenol red and glucose were dispensed into PM-M TOX1 MicroPlate (Biolog, Hayward, CA) wells whose rows contain different carbon-energy sources as indicated. After 48 h incubation, PM-M1, PM-M2, PM-M3, and PM-M4 plates were incubated at 37 ^o^C under air to assess dye reduction for 6 h (Redox Dye Mix MA) and then photographed. For PM-M TOX1 MicroPlates, Biolog Redox Dye Mix MA without or containing glucose was added after two days of incubation, plates were incubated at 37 ^o^C under air to assess dye reduction 6 h and then photographed. The 48-h incubation should allow cells to use up residual carbon-energy sources in the 5% serum (5% serum would contribute about 0.35 mmol/L glucose, plus lipids, and amino acids) and minimizes the background color in the negative control wells, which have no added biochemical substrate. Furthermore, the 48-h incubation should allow cells to transition their metabolism to use the various substrates provided in the wells. The respective utilization of substrates to generate energy-rich NADH was measured as OD at 590 nm. Negative controls (red boxes in Figure 1) have no substrate in the well. Wells containing D-glucose (green boxes in Figure 1) served as positive controls. Thresholds were set to disregard small and insignificant changes, and all wells that exceeded this threshold were considered to denote differentially-metabolized substrates for each cell line. Parallel experiments using conventional 96-well plates were performed using the MTT (3–4, 5-dimethylthiazol-2-yl-2, 5-diphenyl-tetrazolium bromide) redox assay to ensure that the 48-h incubation period with Biolog IF-M1 medium failed to significantly alter the baseline cell growth of MCF10A R132H/+ and MCF10A IDH1 +/+ cells (data not shown).

### Cell viability assays

The cell viability effects of metformin, BPTES, AGI-5198, as single agents or combined as specified, were determined using the standard colorimetric MTT reduction assay. For each treatment, cell viability was evaluated as a percentage using the following equation: (OD_570_ of the treated sample/OD_570_ of the untreated sample) × 100.

### Cell proliferation assays

Semi-confluent cultures of MCF10A R132H/+ and MCF10A IDH1 +/+ cells were trypsinized and seeded in 24-well plates at a density of 5000 cells/well. Cells were incubated for 18 h to allow attachment, after which a 0-time point measurement was determined. Cells were then cultured in standard medium containing 5% serum in the absence or presence of graded concentrations of metformin and counted at days 2, 4, 6, and 8 using a Coulter Counter (Coulter Electronics, Inc.). All assays were performed at least three times in duplicate.

### Colony formation assays

Anchorage-dependent clonogenic growth assays were performed by initially seeding MCF10A R132H/+ and MCF10A IDH1 +/+ cells into 100-mm plates at low density. Cells were either left untreated or treated for 48 hours with graded concentrations of metformin (0.1, 1, and 10 mmol/L). Cells were then replated in six-well plates (approx. 100 cells/well) and cultured in drug-free medium for 7 to 10 days in a humidified atmosphere with 5% CO_2_, at 37°C. Colonies were stained with crystal violet and the number of colonies with > 50 cells were counted.

### Mammosphere culture

Mammospheres were generated from single cells of MCF10A R132H/+ and MCF10A IDH1 +/+ cell lines seeded at 1000 cells/cm^2^ in six-well ultralow attachment plates (Corning Inc.). Mammosphere medium consisted of F-12/DMEM containing 5 mg/mL insulin, 0.5 mg/mL hydrocortisone, 2% B27 supplement (Invitrogen Ltd.), and 20 ng/mL epidermal growth factor. The medium was made semi-solid by the addition of 0.5% methylcellulose (R&D Systems, Minneapolis, MN) to prevent cell aggregation.

### Mammosphere-forming efficiency

Mammosphere-forming efficiency (MSFE) was calculated as the number of sphere-like structures (diameter >50 μm) formed after 7 days in the absence or presence of 1 and 10 mmol/L metformin, divided by the original number of cells seeded and expressed as a percentage (mean ± SD).

### Targeted metabolomics

We employed a method based on gas chromatography coupled to a quadrupole time-of-flight mass spectrometer and an electron impact interface (GC-EI-QTOF-MS), allowing the identification and quantification of 22 metabolites (Figure 4). To minimize complexity in the metabolite extraction, we used methanol mixed with D4-succinic acid as surrogate standard (MeOH-D_4_S) to obtain a final concentration of 0.01 μmol/L. Cell pellets were resuspended in 500 μL of MeOH-D_4_S and lysed with three cycles of freezing and thawing using liquid N_2_ and sonicated with three cycles of 30 seconds. Samples were maintained in ice for 1 minute between each sonication step. Proteins were precipitated, samples were centrifuged and supernatant was collected. After metabolite extraction, samples were dried under N_2_ and derivatized to rapidly form silyl derivatives. Briefly, we added 30 μL of methoxylamine hydrochloride dissolved in pyridine (40 mg/mL) to each sample, which was incubated for 1.5 hours at 37°C with agitation. Forty-five μL of N-methyl-N-(trimethylsilyl)-trifluoroacetamide (TMS) was then added to each sample, which were agitated for 10 minutes and placed in darkness for 1 hour before immediate analysis. Raw data were processed and compounds were detected and quantified using the Qualitative and Quantitative Analysis B.06.00 software (Agilent Technologies), respectively. Note: A detailed description of this procedure is given in: Marta Riera-Borrull, Esther Rodríguez-Gallego, Anna Hernández-Aguilera, Rosa Ras, Elisabet Cuyàs, Jordi Camps, Angel G, Corbí, Antonio Segura-Carretero, Javier A. Menendez, Jorge Joven, Salvador Fernández-Arroyo. *Exploring the process of energy generation in pathophysiology by targeted metabolomics: Performance of a simple and quantitative method* (manuscript in preparation).

### Statistical analysis

Cell proliferation and cell viability results are presented as the mean±SD for at least three repeated individual experiments for each group. Two-group comparisons were performed by Student's *t* test for paired and unpaired values. In all cases, statistical analysis was carried out with XLSTAT (Addinsoft™) and *P* < 0.05 and *P* < 0.01 were considered to be significant. Results from targeted metabolomics were compared by one-way ANOVA with Dunnett's multiple pair-wise comparison tests using a significance threshold of 0.05. Other calculations including comparisons with the U of Mann-Whitney test and/or correlations were made using GraphPad Prism software 6.01 (GraphPad Software, San Diego, CA, USA).
